# Topology, Antiviral Functional Residues and Mechanism of IFITM1

**DOI:** 10.3390/v12030295

**Published:** 2020-03-08

**Authors:** Fang Sun, Zhiqiang Xia, Yuewen Han, Minjun Gao, Luyao Wang, Yingliang Wu, Jean-Marc Sabatier, Lixia Miao, Zhijian Cao

**Affiliations:** 1 State Key Laboratory of Virology, Modern Virology Research Center, College of Life Sciences, Wuhan University, Wuhan 430072, China; 2016202040055@whu.edu.cn (F.S.); 2016202040013@whu.edu.cn (Z.X.); 2017282040174@whu.edu.cn (Y.H.); MinjunGao@Whu.edu.cn (M.G.); 2018202040036@whu.edu.cn (L.W.); ylwu@whu.edu.cn (Y.W.); 2 Institute of NeuroPhysiopathology, UMR 7051, Faculté de Médecine Secteur Nord, 51, Boulevard Pierre Dramard, CS80011, CEDEX 15, 13344 Marseille, France; sabatier.jm1@libertysurf.fr; 3 Department of Biochemistry, School of Basic Medical Sciences, Wuhan University, Wuhan 430071, China; miaolixia@whu.edu.cn; 4 Hubei Province Engineering and Technology Research, Center for Fluorinated Pharmaceuticals, Wuhan University, Wuhan 430072, China

**Keywords:** IFITM1, topology, antiviral functional residues, mechanism

## Abstract

Interferon-inducible transmembrane proteins (IFITM1/2/3) have been reported to suppress the entry of a wide range of viruses. However, their antiviral functional residues and specific mechanisms are still unclear. Here, we firstly resolved the topology of IFITM1 on the plasma membrane where N-terminus points into the cytoplasm and C-terminus resides extracellularly. Further, KRRK basic residues of IFITM1 locating at 62–67 of the conserved intracellular loop (CIL) were found to play a key role in the restriction on the Zika virus (ZIKV) and dengue virus (DENV). Similarly, KRRK basic residues of IFITM2/3 also contributed to suppressing ZIKV replication. Finally, IFITM1 was revealed to be capable of restricting the release of ZIKV particles from endosome to cytosol so as to impede the entry of ZIKV into host cells, which was tightly related with the inhibition of IFITM1 on the acidification of organelles. Overall, our study provided topology, antiviral functional residues and the mechanism of interferon-inducible transmembrane proteins.

## 1. Introduction

Human interferon-induced transmembrane protein 1/2/3 (IFITM1/2/3) is widely expressed in different tissues and organs, and the expression level of these proteins is regulated by interferon [1]. They contain 125–133 amino acid residues and have two transmembrane domains. The topologies of IFITM1, IFITM2 and IFITM3 are similar. They all consist of five parts, including short N-terminal domain (NTD) and C-terminal domain (CTD), intramembrane domain 1 (IM1) and intramembrane domain 2 (IM2), and short conserved intracellular loop (CIL) [2]. At least three possible topological models of IFITMs have been reported [3]. One model is that N-terminus and C-terminus reside extracellularly or in intracavity, IM1 and IM2 stretch across the phospholipid bilayer and CIL resides in the cytoplasm. The second model is that N-terminus, C-terminus, and CTD are in the cytoplasm, and IM1 and IM2 are in the phospholipid bilayer. The third model is that N-terminus and CIL reside in the cytoplasm, and CTD resides extracellularly or in intracavity [3]. Therefore, the exact topology of IFITMs is still to be investigated.

IFITM proteins are involved in the regulation of many activities, such as tumorigenesis [4] and immune signal transduction [5]. Most importantly, it has been reported that IFITM proteins have a broad antiviral spectrum in recent decades. IFITM proteins can effectively block a variety of enveloped viruses from invading host cells, such as influenza A virus (IAV) of Orthomyxoviridae [6], respiratory syncytial virus (RSV) of Pneumoviridae [7], ZIKV and DENV of Flaviviridae [8,9], ebolavirus (EBOV) and severe acute respiratory syndrome virus (SARS-CoV) of [10]. On the other hand, IFITM proteins also restrict some nonenveloped viruses, such as reovirus (ROV) [11]. Additionally, IFITMs have almost no inhibitory effect on certain viral infections, such as adenovirus (AdV) [12] and Sendai virus (SeV) [13]. Although IFITMs play critical antiviral roles in the innate immune system, their antiviral functional residues are mostly still unclear. There is a report showing IM1 of IFITM3 is required for both the interaction of IFITM protein and the restriction of IAV and DENV [9]; therefore, vital antiviral functional residues of IFITM1 are to be determined.

IFITM-mediated restriction on viruses mainly occurs at the stage of virus invasion into host cells [14]. Some studies suggest that IFITM proteins may suppress the entry of viruses by inhibiting the hemifusion of viral membrane and host cell membranes [15] or restricting the formation of fusion pores following virus-endosome hemifusion [16]. There is also a finding that IFITM3 disrupts intracellular cholesterol homeostasis to block viral release into the cytosol by interacting with vesicle–membrane–protein-associated protein A [17]; clearly, the antiviral mechanisms of IFITMs are extremely diverse and complex, and still need to be studied.

In our study, immunofluorescence and confocol microscopy analysis showed that IFITM1 was mainly distributed on the plasma membrane adopting the topological model that N-terminus of IFITM1 pointed into the cytoplasm and C-terminus extended extracellularly. Alanine scanning and site-directed mutations found that KRRK basic residues were key for the restriction of IFITM proteins on ZIKV and DENV. By electron microscope and flow cytometry analysis, IFITM1 was revealed to restrict the release of ZIKV from endosome to cytosol, which was tightly related to its inhibition against the acidification of organelles.

## 2. Materials and Methods

### 2.1. Cells

HEK293A, HEK293T, Huh7 and Vero cells were purchased from the China Center for Type Culture Collection (CCTCC). All cells were cultured in DMEM (Gibco-Invitrogen, New York, NY, USA) supplemented with 10% FBS (Gibco-Invitrogen) and 1% penicillin/streptomycin at 37 °C in an incubator filled with 5% CO_2_.

### 2.2. Antibodies and Reagents

Zika virus envelope (E) protein antibody (GTX133314) was purchased from GeneTex (San Antonio, USA). Dengue virus type 1–4 antibody (MA1-27093) was purchased from Invitrogen (Shanghai, China). Abs against Flag (F1804) and Myc (A00863-100) were purchased from Sigma–Aldrich (Shanghai, China) and GenScript (Nanjing, China), respectively. Abs against HA (66006-2-lg) and GAPDH (60004-1-lg) were purchased from ProteinTech Group (Wuhan, China). Anti-sodium potassium ATPase (Na^+^/K^+^-ATPase) antibody (EP1845Y) was kindly offered by Jing Yao from the College of Life Sciences at Wuhan University. Alexa Fluor 488 (34106ES60) and Alexa Fluor 647 AffiniPure Donkey Anti-Mouse (34113ES60) were purchased from YEASEN (Shanghai, China). LysoSensor™ Green DND-189 (L7535) and Thermo Fisher Scientific Page Ruler Prestained Protein Ladder (26617) were purchased from Invitrogen (Shanghai, China). Bestar^®^ SYBR Green qPCR master mix reagent (DBI-2073) was purchased from DBI^®^ Bioscience (Shanghai, China).

### 2.3. Plasmids and Transfection

pcDNA3.1-Flag-IFITM1/IFITM2/IFITM3, pcDNA3.1-Flag-IFITM-mutants, pcDNA3.1-HA-IFITM1-Myc, pt-Dimer-Rab5 and pt-Dimer-Rab7 were constructed by our laboratory. pEGFP-LAMP1 was kindly offered by Hongbing Shu and Zhiyin Song from the College of Life Sciences, Wuhan University. When covering about 70% of the culture flask, cells were transfected with recombinant plasmids using TurboFect transfection reagent (Invitrogen, R0531) following the instructions; then, cells were cultured for 24 h.

### 2.4. Viruses and Infection

ZIKV Puerto Rico strain (PRVABC59) cDNA plasmid was kindly provided by Ren Sun and Danyang Gong at UCLA. DENV serotype 2 TSV01 strain (DENV-2 TSV01) was kindly provided by Bo Zhang from Wuhan Institution of Virology, Chinese Academy of Sciences. SeV and AdV were kindly supplied by Mingxiong Guo and Zan Huang from the College of Life Sciences at Wuhan University, respectively.

After transfecting IFITM plasmid for 24 h, cells were incubated with ZIKV (MOI = 0.1), DENV (MOI = 0.1), SeV (MOI = 0.1) and AdV (MOI = 1) for 1 h at 37 °C, respectively. Then, the supernatant was removed and the cells were cultured with fresh DMEM supplemented with 10% FBS and 1% penicillin/streptomycin for 48 h. Next, cells were collected to be detected.

### 2.5. Confocal Microscopy

Cells were fixed with precooled 4% paraformaldehyde for 15 min at room temperature followed by washing them in PBS twice (5 min each time). Then, samples were treated without 0.2% Triton X-100 (intact cell membranes) or with 0.2% Triton X-100 (permeabilized cell membranes) for 8 min followed by washing them in PBS twice (5 min each time). Then, nonspecific proteins were blocked for 45 min with 5% bovine serum albumin (BSA) in PBS. Next, cells were incubated with primary antibody at 4 °C overnight and washed three times in PBS (10 min each time). Later, cells were incubated with Alexa Fluor-labeled secondary antibody for 2 h at room temperature followed by washing them in PBS three times for 10 min each time. Cell nuclei were subsequently stained with DAPI Fluoromount-G^®^ (antGene, Wuhan, China, ANT072). The fluorescence was observed using a confocal laser-scanning microscope (Leica TCS SP2, Wetzlar, Germany).

### 2.6. Western Blotting

Cells were lysed with 1% SDS. Samples were boiled in boiling water for 20 min and centrifuged at 12,000 rpm for 10 min at 4 °C to remove cell debris. The total protein content of the supernatants was measured using a BCA protein assay kit (ThermoFisher Scientific, Shanghai, China) and was mixed with 5× loading sample buffer (Biosharp, BL502A, Hefei, China). Then, samples were boiled in boiling water for 10 min. Equal amounts of proteins were separated by 10% SDS-polyacrylamide gel electrophoresis (PAGE) and transferred to nitrocellulose membranes (NC membrane, Millipore, Wuhan, China). NC membranes were incubated with 5% nonfat milk for 2 h at room temperature to block nonspecific proteins. Then, NC membranes were incubated with primary antibodies at 4 °C overnight, followed by incubation with secondary antibodies for 2 h at room temperature. Antibodies were used following the instructions. Later, NC membranes were detected by chemiluminescence using a WesternBrightTM ECL Western blotting detection kit (Advansta, Menlo Park, USA).

### 2.7. Quantitative Real-Time PCR Assay (qPCR)

mRNA of cells was extracted with Trizol reagent (Takara, Beijing, China), and the first-strand cDNA was reversed-transcribed by using the Revert Aid first-strand cDNA synthesis kit (Thermo Scientific). The cDNA was quantitated by qPCR with SeV and GAPDH primers using the Bestar^®^ SYBR Green qPCR master mix reagent (DBI^®^ Bioscience, Shanghai, China). The primers of ZIKV, SeV and GAPDH were synthesized as our laboratory previously described [18]. qPCR experiments were performed on an ABI 7500 system (Shanghai, China) according to the instructions.

### 2.8. Flow Cytometry

Cells were incubated with 1 μM LysoSensor DND-189 at 37 °C for 20 min. Then, cells were digested with 0.25% trypsin and passed through nylon net to obtain a single-cell suspension. Flow cytometry measuring the intensity of FITC was performed on a Cytoflex flow cytometer. If IFITM1 and 1-KRRK alkalize acidic organelles, the peak of FITC would shift to the left relative to the negative control.

### 2.9. Electron Microscopy (EM)

Vero cells were transfected with pcDNA3.1, pcDNA3.1-Flag-IFITM1 and pcDNA3.1-Flag-1-KRRK in a 6 cm dish, respectively. After 24 h, cells were incubated with ZIKV (MOI = 10) at 4 °C for 4 h, followed by being cultured for 2 h at 37 °C in an incubator filled with 5% CO_2_. The supernatant was removed and then cells were fixed with 5% glutaraldehyde at 4 °C for 15 min. Cells were collected and centrifuged at 2000 rpm for 5 min. After that, cells were treated with 5% glutaraldehyde at 4 °C for 4 h to be detected. EM samples were commissioned to Wuhan Institution of Virology, Chinese Academy of Sciences, to prepare and observe. Samples were examined with a Tecnai EM with an accelerating voltage of 200 kV and magnifications of ×11,500 and ×5000.

### 2.10. Statistical Analysis

Adobe Photoshop CS6 and Graphpad Prism6 were used for statistical analysis. Data were usually representative and presented as the mean ± standard deviation (SD) of the three independent experiments. *p* values were calculated by the Student t-test. Statistical significance was considered at *p* value less than 0.05 (* < 0.05, ** < 0.01 and *** < 0.001).

## 3. Results

### 3.1. IFITM1 Distributes Widely on Plasma Membrane and Cytoplasm

**Figure 1:**
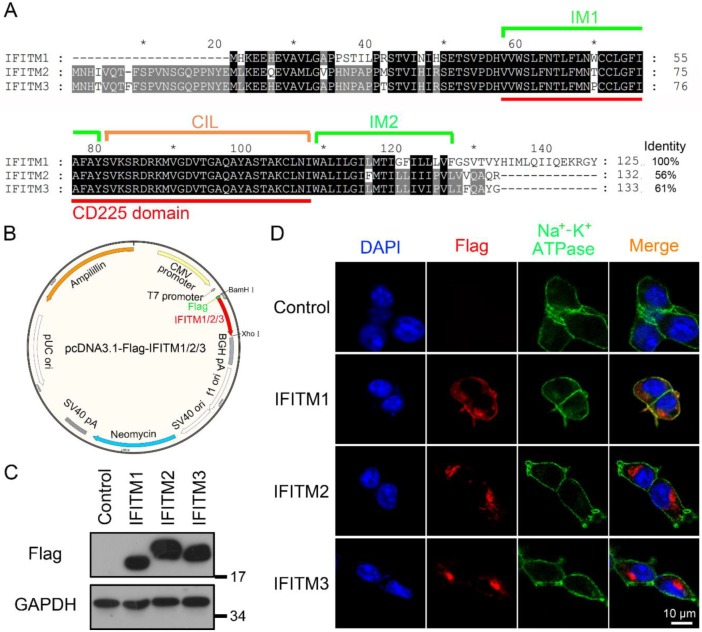
The subcellular localization of IFITM1/2/3 in HEK293T cells. (**A**) The amino acid sequence alignment of IFITM1, IFITM2 and IFITM3. Predicted intramembrane domain 1 (IM1) and intramembrane domain 2 (IM2; green line), conserved intracellular loop (CIL; orange line) and CD225 domain (red line). (**B**) The plasmid map of pcDNA3.1-Flag-IFITM1/2/3. The map was drawn with SnapGene. (**C**) The overexpression analysis of Flag-IFITM1/2/3 in HEK293T cells; pcDNA3.1 and pcDNA3.1-Flag-IFITM1/2/3 were transfected in HEK293T cells, respectively. After 24 h, cells were collected, and the expression level of intracellular IFITM1/2/3 was analyzed by Western blotting using anti-Flag antibody. (**D**) The colocalization of IFITM1/2/3 and Na^+^-K^+^ ATPase in HEK293T cells; pcDNA3.1 and pcDNA3.1-Flag-IFITM1/2/3 were transfected in HEK293T cells. After 24 h, cells were stained with anti-Flag antibody, anti-Na^+^-K^+^ ATPase antibody and DAPI. Then, cells were observed using confocal microscopy. Flag-IFITM1/2/3, red; Na^+^-K^+^ ATPase, green; DAPI, blue.

IFITM proteins widely expressed in different tissues and organs were found to play important antiviral roles in the innate immune system. Although they share a high identity of amino acid sequences (Figure 1A) and are able to inhibit a wide range of viruses, IFITM1/2/3 still have different antiviral activities and spectra. To determine if the intracellular distribution of IFITM proteins influences their antiviral activities, we analyzed their intracellular location. The cDNA sequences of IFITM1/2/3 were cloned from HEK293T cells by RT-PCR and then were inserted into pcDNA3.1 plasmid with BamHI and XhoI, where a Flag-tag was added to their N-terminus (Figure 1B). The overexpression level of pcDNA3.1-Flag-IFITM1/2/3 was detected, and the result showed that they were greatly expressed compared with the control (Figure 1C). Na^+^/K^+^-ATPase is a biomarker of the plasma membrane [19]. We analyzed the colocalization of IFITM1/2/3 and Na^+^-K^+^ ATPase by immunofluorescence using the anti-Flag antibody and Na^+^/K^+^-ATPase antibody coupled with Alexa Fluor^®^ 488. The experimental data showed that IFITM1 distributed on the plasma membrane and in the cytoplasm. IFITM1 especially had strong colocalization with Na^+^-K^+^ ATPase (Figure 1D). In comparison, IFITM2 and IFITM3 appeared to reside only in cytoplasm (Figure 1D). These data indicate IFITM1 distributes widely on the plasma membrane and cytoplasm.

### 3.2. IFITM1 Adopts the Topology on Plasma Membrane Where N-Terminus Points into the Cytoplasm and C-Terminus Resides Extracellularly

**Figure 2:**
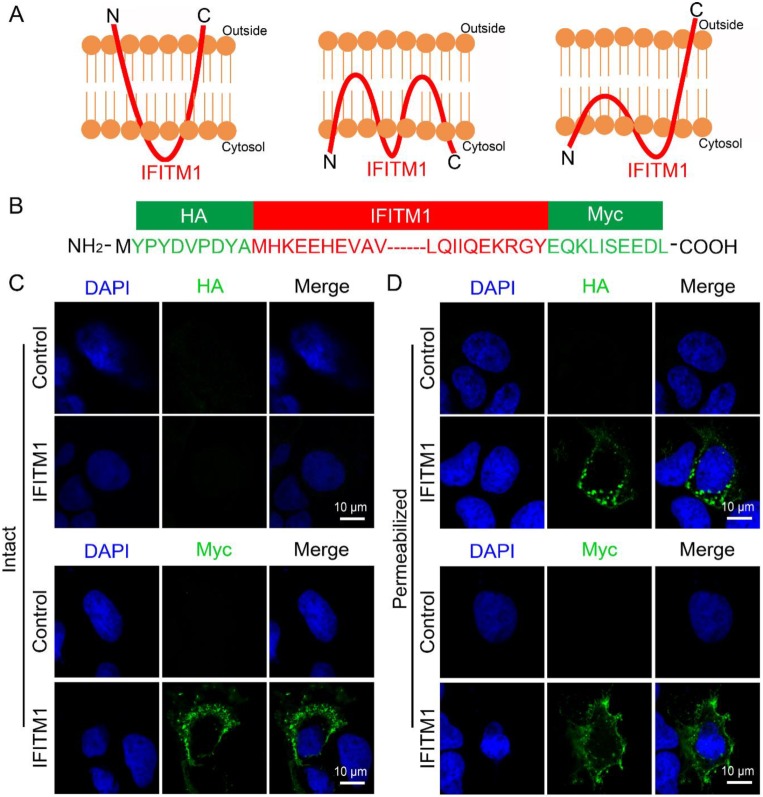
The topological structure analysis of IFITM1 on cell membranes. (**A**) Three different topological models of IFITM1 in cell membranes. IFITM1 was drawn using the red curve. N and C represented N-terminus and C-terminus, respectively. (**B**) Sketch of the amino acid sequence of HA-IFITM1-Myc. (**C**,**D**) The location of HA-IFITM1-Myc in Huh7 cells. pcDNA3.1 and pcDNA3.1-HA-IFITM1-Myc were transfected in Huh7 cells, respectively. After 24 h, cells were treated without 0.2% Triton X-100 (**C**) or with 0.2% Triton X-100 (**D**). Then, cells were stained with anti-HA antibody or anti-Myc antibody and DAPI. Cells were observed using confocal microscopy. HA-IFITM1-Myc, green. DAPI, blue.

Previously, IFITMs have been reported to have three topological models on the cell membranes (Figure 2A) [20]. To determine the detailed topological model that IFITM1 adopted on the cell membranes, we constructed pcDNA3.1-HA-IFITM1-Myc plasmid to express IFITM1 protein fused N-terminal HA-tag and C-terminal Myc-tag for the experiments. The sketch of the amino acid sequence of HA-IFITM1-Myc is shown in Figure 2B. The location of HA-IFITM1-Myc was analyzed using anti-HA antibody or anti-Myc antibody in Huh7 cells when the plasma membranes were intact or permeabilized. We found that HA-IFITM1-Myc could not be dyed using anti-HA antibody when plasma membranes were intact, but it could be observed when cell membranes were permeabilized (Figure 2C,D), which suggested that the N-terminus of IFITM1 points into the cytoplasm. Differently, HA-IFITM1-Myc could be observed using anti-Myc antibody when cells were treated with 0.2% Triton X-100 or not (Figure 2C,D), which indicated that the C-terminus of IFITM1 was located outside the plasma membranes. Similarly, the location of HA-IFITM2/3-Myc was analyzed using anti-HA antibody or anti-Myc antibody in Huh7 cells when plasma membranes were intact or permeabilized. We found that neither HA-IFITM2-Myc or HA-IFITM3-Myc could be dyed using anti-HA antibody or anti-Myc antibody when plasma membranes were intact, but both of them could be observed when cell membranes were permeabilized (Figure S1). These results indicated that IFITM2 and IFITM3 are also located in the cytoplasm of Huh7 cells.

These data show that IFITM1 adopts the topological structure (Figure 2A, right) on the plasma membranes, where the N-terminus points into the cytoplasm and the C-terminus resides extracellularly, consistent with the topology model that NTD and CIL are in the cytoplasm and CTD is extracellular or intracavity.

### 3.3. KRRK Basic Residues of IFITM1 Are Key for the Restriction on ZIKV and DENV

**Figure 3:**
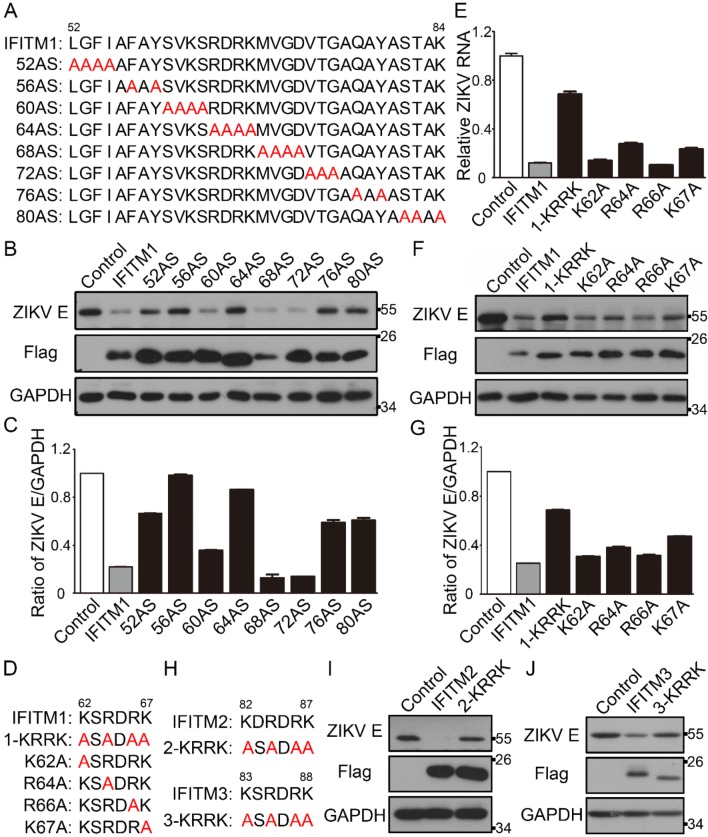
The antiviral functional residues analysis of IFITM1/2/3 against ZIKV. (**A**) Eight alanine scan (AS) mutants of IFITM1. Each AS mutant is named for the first of four sequential residues that have been substituted with alanines (marked red). (**B**,**C**) The inhibitory effect of IFITM1 and AS mutants of IFITM1 on ZIKV replication. pcDNA3.1, pcDNA3.1-Flag-IFITM1 and AS mutants of pcDNA3.1-Flag-IFITM1 were transfected in Vero cells, respectively. After 24 h, cells were infected with ZIKV (MOI = 0.1). After 48 h, cells were collected, and the intracellular expression level of ZIKV E protein was analyzed by Western blotting (**B**). Grayscale of figure B was analyzed with Image J software (**C**). (**D**) The KRRK mutant and single point mutants of IFITM1. (**E**–**G**) The inhibitory effect of IFITM1 and mutants of IFITM1 on ZIKV replication. pcDNA3.1, pcDNA3.1-Flag-IFITM1 and mutants of pcDNA3.1-Flag-IFITM1 were transfected in Vero cells, respectively. After 24 h, cells were infected with ZIKV. After 48 h, cells were collected, and the relative intracellular ZIKV RNA was analyzed by qPCR (**E**), and the expression level of intracellular ZIKV E protein was analyzed by Western blotting (**F**). (**G**) Grayscale of figure F was analyzed with Image J software. (**H**) The KRRK mutants of IFITM2 and IFITM3. (**I**,**J**) The inhibitory activity of IFITM2/3 and their mutants against ZIKV replication. pcDNA3.1-Flag-IFITM2 and mutant of pcDNA3.1-Flag-IFITM2 (**I**), pcDNA3.1-Flag-IFITM3 and mutant of pcDNA3.1-Flag-IFITM3 (**J**), were transfected in Vero cells, respectively. After 24 h, cells were infected with ZIKV. After 48 h, cells were collected and the intracellular expression level of ZIKV E protein was analyzed by Western blotting.

An amphipathic sequence of amino acids (residues 38–47) of IFITM1 has been reported to be required for the restriction on H1N1 IAV [21]. However, the other antiviral functional residues of IFITM1 are still unclear. Therefore, eight alanine scan (AS) mutants of the IFITM1 CD225 domain were constructed and named (Figure 3A). Vero cells were kidney cells of the monkey *Chlorocebus sabaeus* from Africa, which was easily infected by ZIKV [22]. We observed the location of IFITM1/2/3 fused with a Flag-tag in Vero cells; the result showed that over-expressed IFITM1 was located both on cell membranes and in the cytoplasm, while IFITM2 and IFITM3 appeared to reside only in the cytoplasm in Vero cells (Figure S2). Then, we compared the inhibitory effects of IFITM1 and eight AS mutants against ZIKV (PRVABC59 strain) in Vero cells. The result showed that IFITM1 inhibited 78% ZIKV E protein compared with the control, while 52AS, 56AS, 60AS, 64AS, 68AS, 72AS, 76AS and 80AS mutants limited 34%, 2%, 65%, 14%, 84%, 86%, 39% and 37% intracellular ZIKV E protein, respectively (Figure 3B,C). Relative intracellular ZIKV E protein was analyzed using ImageJ. These results suggest that 52AS, 56AS, 64AS, 76AS and 80AS mutants of IFITM1 have a weaker antiviral effect than IFITM1 does, and these mutated amino acid residues of IFITM1 may be potential antiviral functional determinants.

To further analyze the key functional antiviral sites of IFITM1, we constructed four single-point mutants of IFITM1 in the CIL domain named K62A, R64A, R66A and K67A and a four-point mutant named 1-KRRK (Figure 3D). Then, we analyzed the antiviral effects of these five mutants. The results showed that 1-KRRK had the least inhibitory activity against ZIKV in Vero cells, and R64A and K67A appeared to have less antiviral effect than the IFITM1 by qPCR (Figure 3E) and Western blotting (Figure 3F,G). However, K62A and R66A still shared similar anti-ZIKV activity with the wild type IFITM1 (Figure 3E–G). The amino acid sequences of IFITM2 and IFITM3 in the CD225 domain were almost the same as those of IFITM1 (Figure 1A). To determine if the KRRK basic residues of IFITM2/3 are also key for their antiviral functions, Flag-IFITM2-KRRK and Flag-IFITM3-KRRK plasmids were constructed. K82, R84, R86 and K87 of IFITM2, and K83, R85, R87 and K88 of IFITM3 were simultaneously mutated to alanine (Figure 3H). Similarly, IFITM2/3 and 2/3-KRRK were used to analyze the antiviral function against ZIKV. We found that both IFITM2 and IFITM3 had a great limitation on the intracellular ZIKV E protein, while 2-KRRK and 3-KRRK had less restriction than their wild types (Figure 3I,J). These data show the KRRK basic residues of IFITM2/3 are also significant to suppress ZIKV infection.

**Figure 4:**
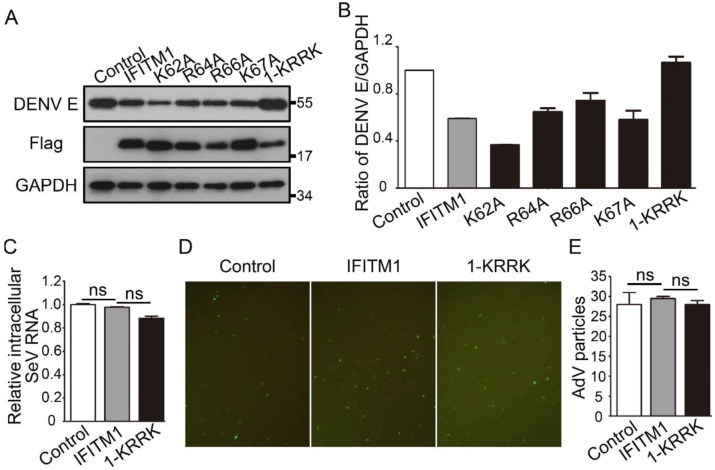
Antiviral functional residues analysis of IFITM1 on dengue virus (DENV), Sendai virus (SeV) and adenovirus (AdV) replications. (**A**,**B**) The inhibitory effect of IFITM1 and mutants of IFITM1 on DENV replication. pcDNA3.1, pcDNA3.1-Flag-IFITM1 and mutants of pcDNA3.1-Flag-IFITM1 were transfected in Vero cells, respectively. After 24 h, cells were infected with DENV (MOI = 0.1). After 48 h, cells were collected, and the expression level of DENV E protein was analyzed by Western blotting (**A**). Grayscale of figure A was analyzed with Image J software (**B**). (**C**) The effect of IFITM1 and 1-KRRK on SeV replication. pcDNA3.1, pcDNA3.1-Flag-IFITM1 and pcDNA3.1-Flag-1-KRRK were transfected in HEK293T cells, respectively. After 24 h, cells were infected with SeV (MOI = 0.1). After 48 h, cells were collected, and intracellular RNA of SeV was analyzed by qPCR. (**D**,**E**) The effect of IFITM1 and 1-KRRK on AdV infection in vitro. pcDNA3.1, pcDNA3.1-Flag-IFITM1 and pcDNA3.1-Flag-1-KRRK were transfected in HEK293A cells, respectively. After 24 h, cells were infected with AdV (MOI = 1). After 48 h, the fluorescence of cells was observed using fluorescence microscopy with the magnification of ×200 (**D**). Fluorescence intensity was counted (**E**). ns = no significance.

DENV, like ZIKV, is a member of the Flavivirus genus. More than 500 million people worldwide are infected each year by any of the four-DENV serotypes [23]. To further verify the importance of the KRRK basic residues of IFITM1 against viruses, IFITM1 and KRRK mutants were used to detect their restriction on the DENV-2 TSV01 strain in Vero cells. The result showed that IFITM1 could decrease the expression of intracellular DENV E protein compared with the control, while 1-KRRK had almost no influence on DENV replication (Figure 4A,B). Therefore, the KRRK basic residues of IFITM1 also played a key role in the restriction on DENV. SeV, a negative single-stranded RNA virus, is a member of the Paramyxoviridae family. AdV is a nonenveloped double-strand DNA virus. We asked whether the KRRK basic residues of IFITM1 had an influence on the infection of SeV and AdV. The results showed that both IFITM1 and 1-KRRK hardly had inhibition on the relative intracellular SeV RNA, compared to the control (Figure 4C). Similarly, they had almost no inhibitory effect on the infection of AdV (Figure 4D,E). In short, the KRRK basic residues of IFITM1/2/3 contributed specifically to their antiviral functions.

**Figure 5:**
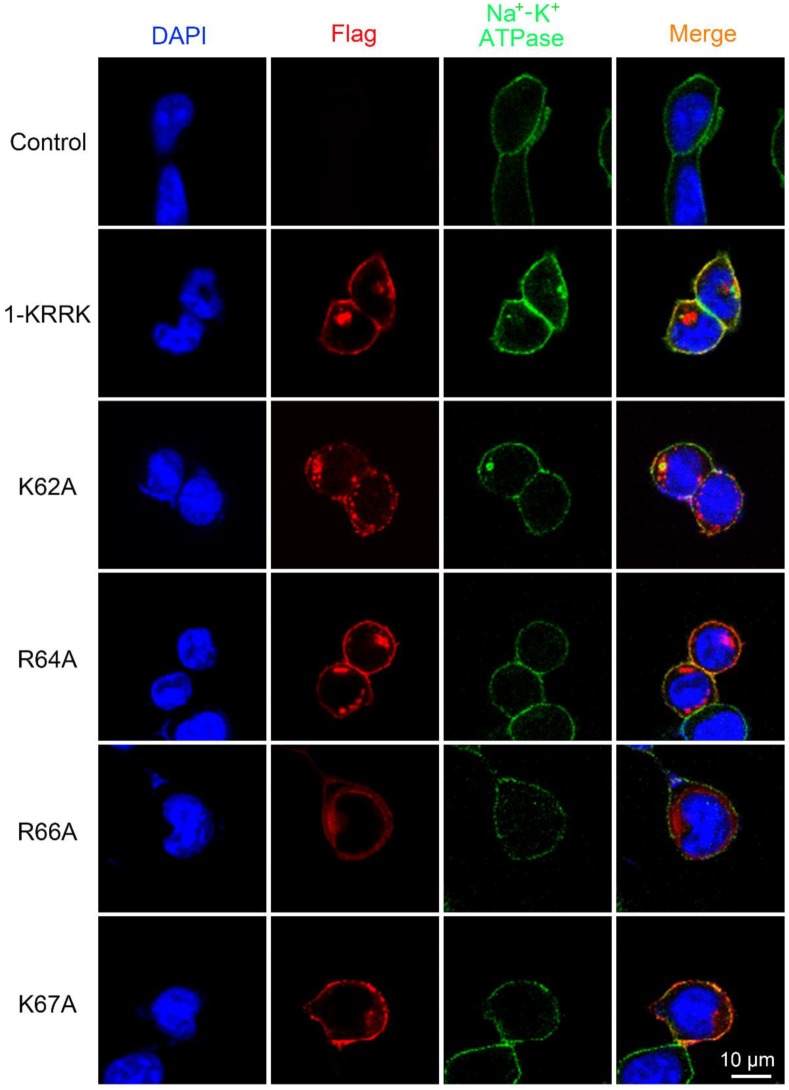
The colocalization analysis of IFITM1 mutants and Na^+^–K^+^ ATPase in HEK293T cells. pcDNA3.1 and pcDNA3.1-Flag-1-KRRK/K62A/R64A/R66A/K67A were transfected in HEK293T cells, respectively. After 24 h, cells were stained with anti-Flag antibody, anti-Na^+^–K^+^ ATPase antibody and DAPI. Then, cells were observed using confocal microscopy. IFITM1 mutants, red; Na^+^–K^+^ ATPase, green; DAPI, blue.

Importantly, we detected the intracellular distributions of five KRRK residues mutants of IFITM1. The results showed 1-KRRK, K62A, R64A, R66A and K67A all had colocalization with Na^+^-K^+^ ATPase in HEK293T cells, and five mutants all could be located in the cytoplasm (Figure 5). In addition, we compared the location of IFITM1 and 1-KRRK in Vero cells. It was found that the localization of 1-KRRK was almost the same as that of IFITM1 (Figure S3). These data indicate these basic amino acids hardly influence the intracellular distribution of IFITM1 and do not explain the reason that the antiviral effect of 1-KRRK is weaker than that of IFITM1.

### 3.4. IFITM1 Restricts the Release of ZIKV from Endosome to Cytosol Related with Inhibiting Organelles Acidification

**Figure 6:**
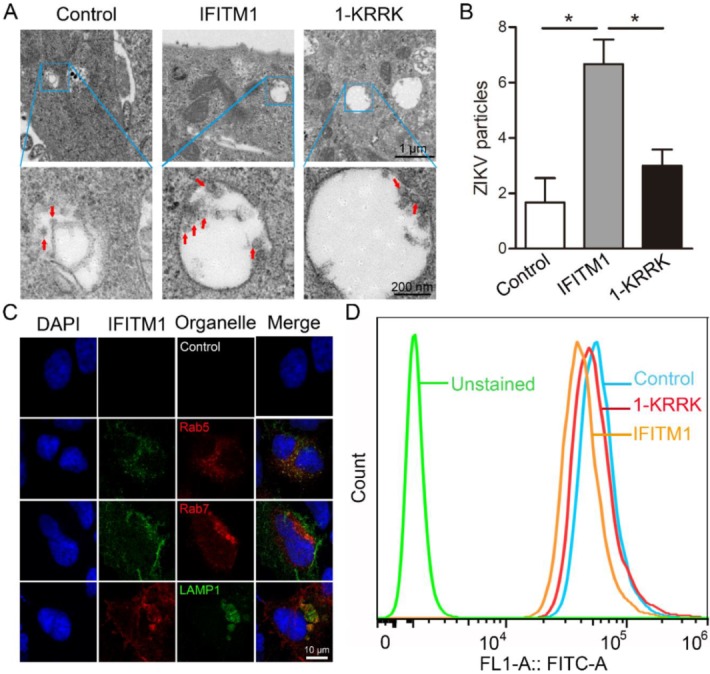
Restriction of IFITM1 on the release of ZIKV from endosome to cytosol by inhibiting the acidification of organelles. (**A**,**B**) The restriction of IFITM1 and 1-KRRK on the release of ZIKV from endosome to cytosol in Vero cells. pcDNA3.1, pcDNA3.1-Flag-IFITM1 and pcDNA3.1-Flag-1-KRRK were transfected in Vero cells, respectively. After 24 h, cells were infected with ZIKV (MOI = 10) and fixed with 5% glutaraldehyde. Cells were observed using electron microscopy (EM) (**A**). Scale bar, 1 μm (up) and 200 nm (down). The ZIKV particles in the endosome were counted (**B**). (**C**) The colocalization of IFITM1 and acidic organelles in Huh7 cells. pcDNA3.1-Flag-IFITM1 and pt-Dimer-Rab5/pt-Dimer-Rab7/pEGFP-LAMP1 were cotransfected in Huh7 cells. After 24 h, cells were stained with anti-Flag antibody and DAPI. Then, cells were observed using confocal microscopy. (**D**) The effect of IFITM1 and 1-KRRK on organelles acidification in Huh7 cells. pcDNA3.1, pcDNA3.1-Flag-IFITM1 and pcDNA3.1-Flag-1-KRRK were transfected in Huh7 cells, respectively. After 48 h, cells were incubated with 1 μM LysoSensor DND-189 at 37 °C for 20 min. The intensity of FITC staining was determined on a Cytoflex flow cytometer. * *p* < 0.05.

ZIKV is a single-stranded RNA virus, and the diameter of its mature viral particles is 40–50 nm [24]. The life cycle of ZIKV can be divided into four stages, including viral entry, genome replication, viral assembly and release [25]. ZIKV enters host cells by clathrin-mediated endocytosis. In this process, the viral envelope and the endosome membrane of host cells are fused under the acidic environment of the endosome; then, the viral genomic RNA is released into the cytoplasm [26]. Previous studies have shown that IFITM proteins can inhibit the entry of ZIKV [8]; however, how IFITM1 inhibits this stage is still unknown. Here, we detected the influence of IFITM1 and 1-KRRK on the release of ZIKV from the endosome to the cytosol. By electron microscopy, we observed IFITM1 prevented more virus particles from being released into the cytoplasm, compared with the negative control, while 1-KRRK virtually unlimited the release of virus particles (Figure 6A). Specific results were counted and shown in Figure 6B, suggesting that IFITM1 did restrict the release of ZIKV from the endosome to the cytosol. In addition, the colocalization of IFITM1 and acidic organelles was detected. Rab5 and Rab7, members of the RAS GTPase superfamily, are mainly located in early endosomes and late endosomes, respectively [27]. LAMP1 is the main constituent protein of the endosome/lysosome pathway [28]. Therefore, we detected the colocalization of IFITM1 and Rab5/Rab7/LAMP1. Indeed, we found that IFITM1 was partially co-located with early endosomes, late endosomes and lysosomes (Figure 6C). This was consistent with previous reports that exogenous IFITM1 can localize on cell membranes and in highly acidified late endosomes and lysosomes [29,30]. We then detected the organelles acidity of Huh7 cells after being over-expressed by IFITM1 and 1-KRRK by a flow cytometer using a 488 nm laser for excitation. Compared with the control group, the curve of IFITM1 shifted to the left, and the curve of 1-KRRK was between IFITM1 and the control (Figure 6D). These data suggested that IFITM1 could inhibit the acidification of organelles, and 1-KRRK had weaker suppressive power than IFITM1 did. Therefore, IFITM1 can restrict the release of ZIKV from endosome to cytosol, which is related with its inhibition on organelles acidification. More importantly, the KRRK basic residues of IFITM1 are vital in these processes.

## 4. Discussion

Previous reports have revealed that IFITM3 adopts the topological model on organelle membranes where its NTD and CIL are in the cytoplasm, and CTD is intracavity by using the combined electron paramagnetic resonance (EPR) and solution nuclear magnetic resonance (NMR) [31]. IFITM1 is high homologous with IFITM3 and has been reported with at least three topological models [2,20]. Here, we support that IFITM1 adopts a topological model on cell membranes where the N-terminus points into the cytoplasm and the C-terminus reside extracellularly. This is consistent with previous reports [2].

The anti-viral function of IFITM proteins was found initially against IAV, West Nile virus (WNV) and DENV in 2009 [32]. Over the next 10 years, IFITM proteins have been reported to restrict many other viruses. ZIKV and DENV are an arthropod-borne virus (arbovirus) in the Flavivirus genus of the Flaviviridae family; both ZIKV and DENV have shown a threat to global health, and the effective vaccines for them still need to be developed [33,34]. IFITM1 is an important downstream gene of interferon; there are reports that show the inhibitory effect of IFITM1 on ZIKV and DENV replication [8,9].

In our study, we conducted a series of experiments to screen the antiviral functional residues of IFITM1 against ZIKV. We found that the KRRK basic residues of IFITM1 were important for the restriction of IFITM1 on ZIKV and DENV. It has been reported that the 80AS and 85AS mutants of IFITM3 have weaker restrictions on IAV and DENV replication than wild type [9]. To further illustrate the importance of these four residues, we also compared the antiviral effects of IFITM2/IFITM3 and their corresponding KRRK mutant. Indeed, the KRRK basic residues of IFITM2/IFITM3 also played a key role in their restriction on ZIKV. Significantly, we found that IFITM1 can restrict the release of ZIKV from endosome to cytosol to prevent the entry of viral particles into host cells, which was associated with its inhibition on organelles acidification. Attractively, 1-KRRK had almost no restriction in this process. Significantly, we found KRRK residues of IFITM1 were very conservative in many species by aligning their amino acid sequences (Figure S4). We hypothesize that KRRK residues of IFITM proteins of other species may play important roles in their antiviral effects.

Most of the previous reports have studied the effect of IFITM3 on viral replication, while our work mainly studied the restriction of IFITM1 on viruses, including ZIKV, DENV, AdV and SeV. Importantly, we found that the KRRK basic residues of IFITM proteins played a vital role in their antiviral processes. In addition, our work provided new insights into the antiviral mechanism of IFITM1 related to inhibiting organelles acidification. The previous research also showed that the endosomes in HULEC cells with a high basal level of IFITM3 were less acidic than in MDCKs [35]. The mechanism may be applicable to explain how IFITM1 restricts other viruses entering cells by clathrin-mediated endocytosis. However, our work could not explain why the KRRK basic residues of IFITM1 are vital for IFITM1 to inhibit viral entry and suppress organelles acidification. The isoelectric point (pI) of IFITM1 with a Flag-tag is 6.2, predicted by the ProtParam website, and the pH values of the late endosome and lysosome are 5.0–5.5 and 4.6–5.0, respectively [36]. Therefore, we hypothesize that IFITM1 may alkalize the acidic organelles by its own properties; besides, the pI of 1-KRRK with a Flag-tag is 5.1 predicted by the ProtParam website, which may explain that 1-KRRK hardly affects the acidification of organelles.

## Supplementary Materials

The following are available online at https://www.mdpi.com/1999-4915/12/3/295/s1. Figure S1. The location of HA-IFITM2/3-Myc in Huh7 cells. Figure S2. The localization analysis of IFITM1/2/3 in Vero cells. Figure S3. The localization analysis of IFITM1/1-KRRK in Vero cells. Figure S4. The amino acid sequence alignment of IFITM1 across nine species.
