# Supplementary material for: Topology, Antiviral Functional Residues and Mechanism of IFITM1
Source: Viruses. 2020 Mar 8;12(3):295. doi: 10.3390/v12030295 (PMC7150853; doi:10.3390/v12030295)
Supplement: Supplementary file 1 [file viruses-12-00295-s001.pdf]

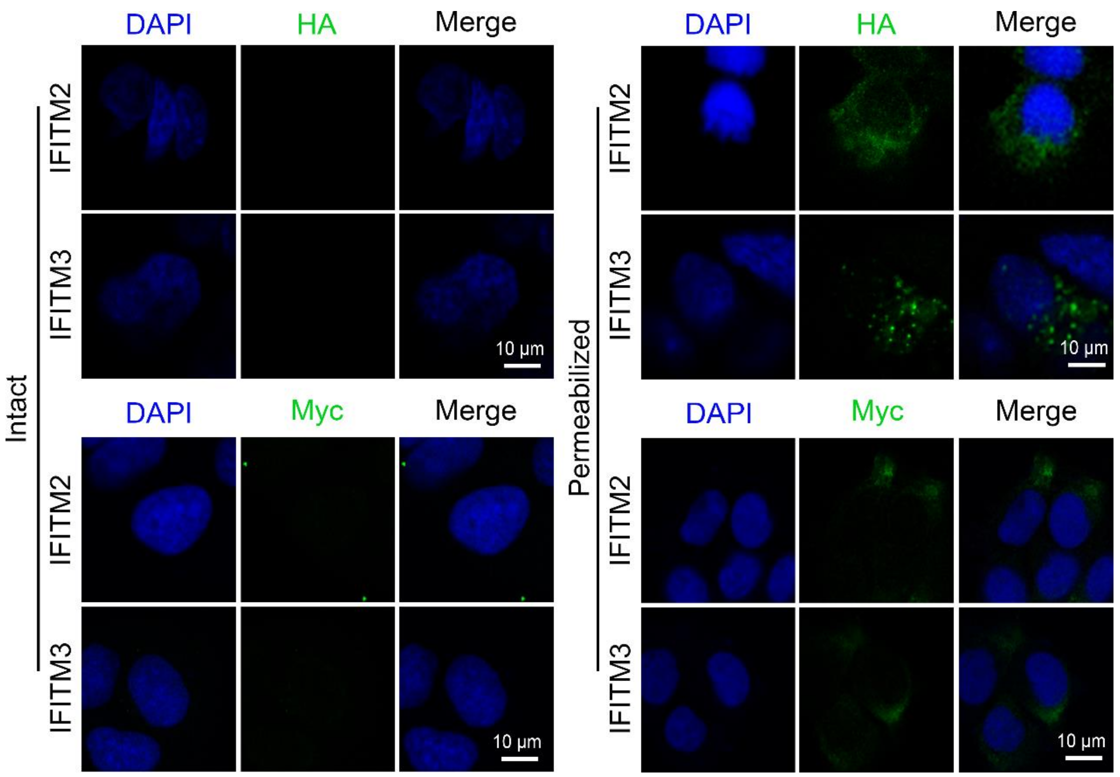

Supplementary Figure 1. The location of HA-IFITM2/3-Myc in Huh7 cells. pcDNA3.1-HA-IFITM2-Myc and pcDNA3.1-HA-IFITM3-Myc were transfected in Huh7 cells, respectively. After 24 h, cells were treated without 0.2% Triton X-100 (left) or with 0.2% Triton X-100 (right). Then, cells were stained with anti-HA antibody or anti-Myc antibody, and DAPI. Cells were observed using a confocal microscopy. HA-IFITM2/3-Myc, green. DAPI, blue.

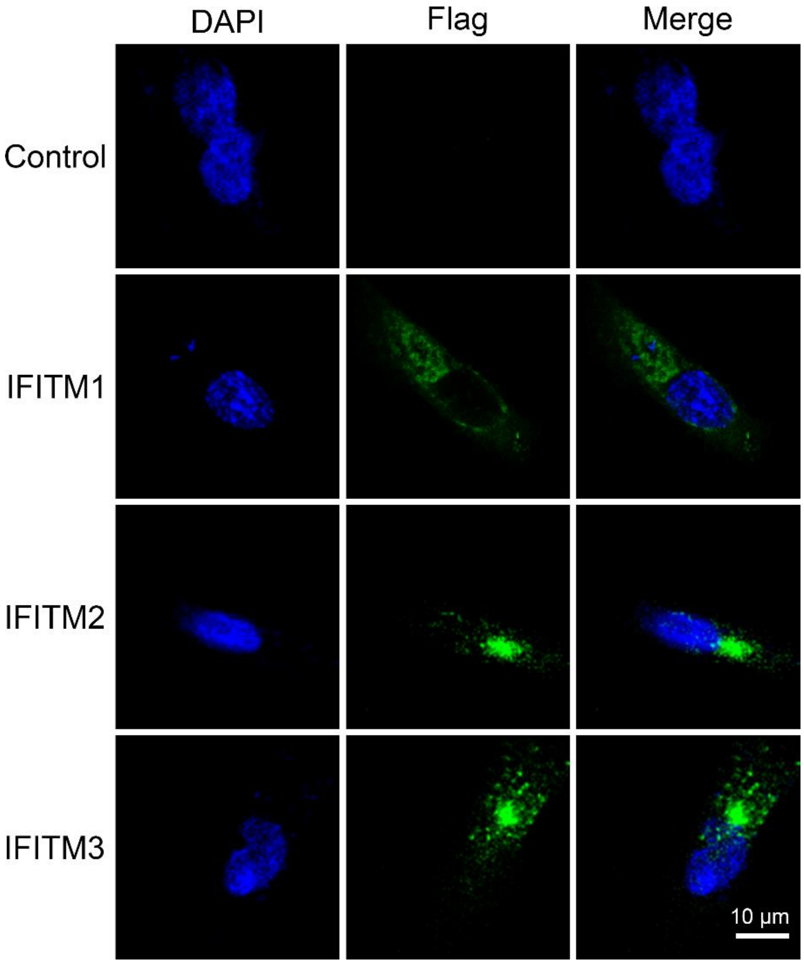

Supplementary Figure 2. The localization analysis of IFITM1/2/3 in Vero cells. pcDNA3.1 and pcDNA3.1-Flag-IFITM1/2/3 were transfected in Vero cells, respectively. After 24 h, cells were stained with anti-Flag antibody, and DAPI. Then, cells were observed using a confocal microscopy. IFITM1/2/3, green. DAPI, blue.

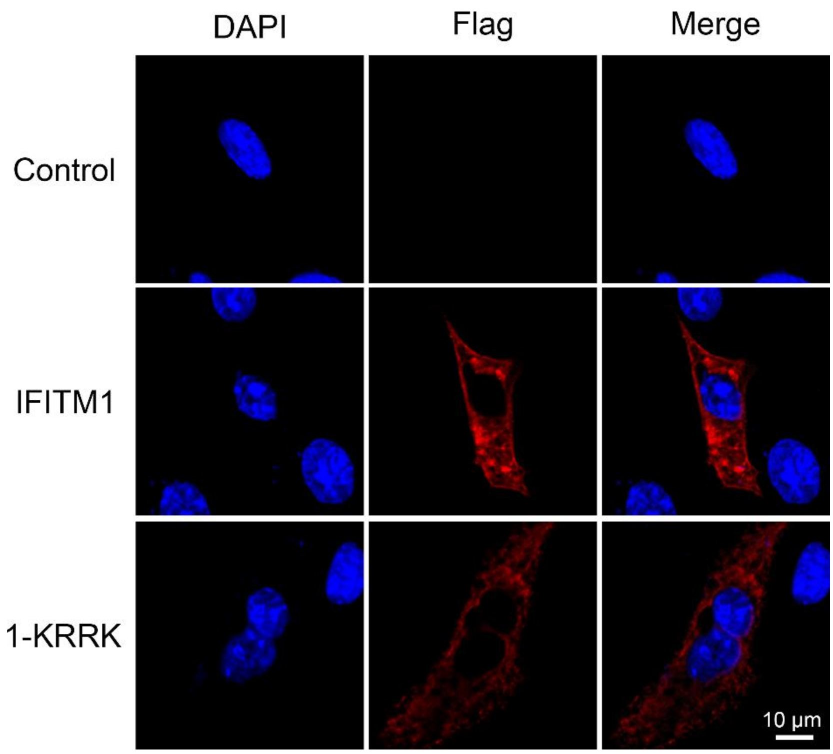

Supplementary Figure 3. The localization analysis of IFITM1/1-KRRK in Vero cells. pcDNA3.1 and pcDNA3.1-Flag-IFITM1/1-KRRK were transfected in Vero cells, respectively. After 24 h, cells were stained with anti-Flag antibody, and DAPI. Then, cells were observed using a confocal microscopy. IFITM1/1-KRRK, red. DAPI, blue.

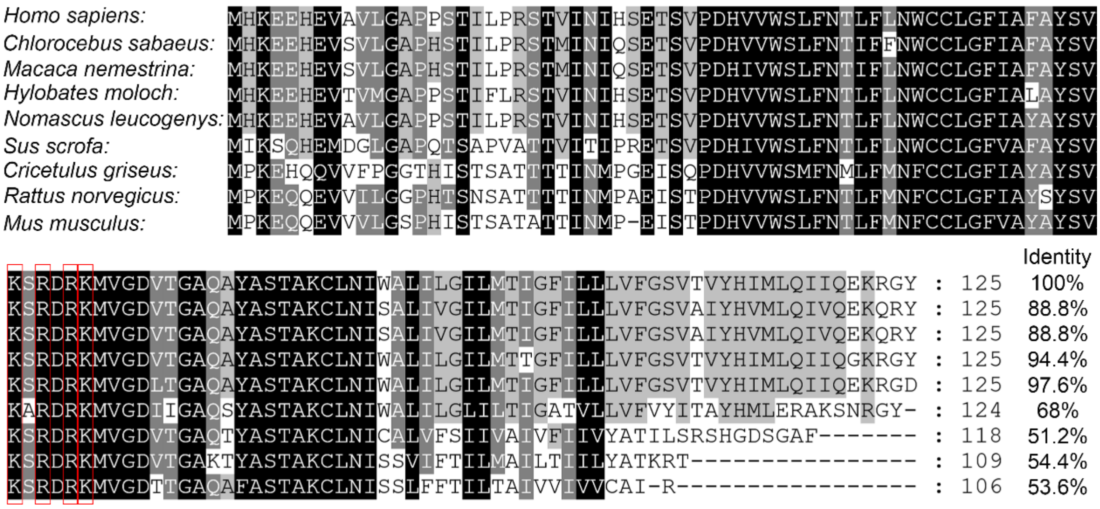

Supplementary Figure 4. The amino acid sequence alignment of IFITM1 across nine species. The amino acid sequences of IFITM1 across nine species were searched for on NCBI. Nine sequences were analyzed and aligned using DNAMAN and GENEDOC software. Two lysines (K) and two arginines (R) were circled with red boxes.
